# Hepatitis C virus genotypes and subtypes circulating in Mainland China

**DOI:** 10.1038/emi.2017.77

**Published:** 2017-11-01

**Authors:** Ying Chen, Changshun Yu, Xueru Yin, Xiaolei Guo, Shangwei Wu, Jinlin Hou

**Affiliations:** 1 Guangzhou Kingmed Center for Clinical Laboratory, Guangzhou, China; 2 College of Laboratory Medicine, Tianjin Medical University, Tianjin, China; 3 Department of Infectious Diseases, Nanfang Hospital, Southern Medical University, Guangzhou, China

**Keywords:** genotyping, hepatitis C virus, subtyping

## Abstract

The hepatitis C virus (HCV) exhibits global genotypic diversity. HCV genotyping plays an important role in epidemiological studies and clinical management. Herein, we report the results of HCV genotype and subtype detection in a large number of clinical samples, as performed by an independent laboratory in China. In total, four HCV genotypes and 18 subtypes were identified among 32 030 patients from 29 provinces and municipalities in China. Five dominant subtypes were detected from 98.84% of the samples: 1b (*n*=16 713, 52.18%), 2a (*n*=9188, 28.69%), 3b (*n*=2261, 7.06%), 6a (*n*=2052, 6.41%) and 3a (*n*=1479, 4.62%). Twelve rare subtypes were detected, of which four (that is, 6b, 6j, 6q and 6r) are reported for the first time in the Chinese population. Genotypes 4, 5 and 7 were not detected. Mixed infections of the dominant subtypes were found in a small portion of samples (*n*=65, 0.203%), in the following combinations: 1b–2a, 1b–3b, 1b–6a, 3a–3b, 1b–3a and 2a–6a. No mixed infections with rare subtypes were found. Males, compared with females, showed higher HCV subtype diversity, a lower percentage of HCV1b and 2a and a higher percentage of rare subtypes and mixed infections. Our analyses revealed the comprehensive distribution patterns of HCV genotypes in the general population of mainland China. HCV genotypic patterns were differentially distributed on the basis of geography, sex and age.

## INTRODUCTION

More than 185 million people have been estimated to be infected with hepatitis C virus (HCV) worldwide, including 350 000 cases of death each year due to infection.^1^ The prevalence of HCV in China has been estimated to be 1%,^2^ thus making the Chinese HCV carrier population(14 million) one of the largest in the world. HCV is a positive-sense, single-stranded RNA (9.6 kb) virus of the *Flaviviridae* family that shows high genetic heterogeneity and can be classified into seven major genotypes (HCV 1–7) including 67 confirmed and 20 provisional subtypes, on the basis of phylogenetic analysis of genome sequences.^3^ Correct determination of HCV genotype is fundamental for evaluation of the epidemiological status of HCV infection and for administration of the most appropriate anti-viral regimen. Furthermore, HCV genotype and subtype are important indicators for anti-viral therapeutic responses.^4, 5^ The Chinese Society of Hepatology and the Chinese Society of Infectious Diseases recommends that HCV genotyping should be done prior to starting anti-viral therapy.^6^

HCV genotype distributions show different geographic patterns.^4^ HCV genotype 1, 2 and 3 have a broad global distribution, whereas genotypes 4, 5 and 6 are seen only in certain areas. For example, genotype 4 is mainly found in the Middle East and North Africa, genotype 5 is seemingly confined to South Africa, genotype 6 is primarily distributed in southern China and South-East Asia,^7^ and genotype 7 has been reported only in central Africa.^8^ Four HCV genotypes (1, 2, 3 and 6) have been reported in China to date, among which subtypes 1b, 2a, 3b, 6a and 3a are the most prevalent subtypes.^9^ Several studies have previously been conducted in China but from relatively small populations and narrow geographical areas; thus, the research findings may not be reliable enough to draw solid conclusions.^10, 11^ Therefore, we conducted an investigation of HCV genotype distributions in a variety of populations from different areas in mainland China. All the tests included in this investigation were performed by a single, independent medical laboratory.

## MATERIALS AND METHODS

### Study population and sample collection

Between January 2013 and January 2017, a total of 56 627 consecutive serum samples were collected for HCV genotyping and subtyping by Kingmed Diagnostics (Guangzhou, Guangdong). Sex and age data corresponding to the samples were analyzed with the test results from this study. For patients with multiple specimens or orders submitted, only the first specimen was analyzed. HCV quantification was also performed at the same time for 734 of the samples.

### TaqMan PCR HCV typing and sanger sequencing confirmation

HCV RNA was extracted using a commercial extraction kit (QIAamp Viral RNA Mini Kit, QIAGEN, Shanghai, China) according to the manufacturer’s instructions. HCV genotyping was performed on all samples by using a TIB HCV TaqMan PCR kit (Triplex International Bioscience, Fuzhou, China), which detects the HCV 1b, 2a, 6a, 3a and 3b subtypes, as approved by the China Food and Drug Administration, according to the manufacturers’ instructions. The low detection limit of the TIB HCV TaqMan PCR kit is ~1000 IU/mL. Samples that failed detection with the TIB HCV TaqMan kit were subsequently analyzed by Sanger sequencing as previously described.^12, 13^ Briefly, two sets of universal primers targeting the core-envelope 1 (C/E1) region of the HCV genome were used for nested PCR amplification and sequencing analysis (Primer set 1: F1, 5′-GCAAC AGGGA ACCTT CCTGG TTGCT C-3′ R1, 5′-CGTAG GGGAC CAGTT CATCA TCAT-3′ Primer set 2: F2, 5′-AACCT TCCTG GTTGC TCTTT CTCTA T-3′, R2, 5′-GTTCA TCATC ATATC CCATG CCAT-3′). The product lengths of primer set 1 and set 2 were 495 and 474 bp, respectively. The subtypes were determined by phylogenic comparison to reference sequences for HCVs, by using MEGA 4.0.^14^

### HCV quantification

Along with HCV genotyping, an HCV quantification assay was also performed on a group of 760 samples with a COBAS AmpliPrep TaqMan kit. The low detection limit of this kit is 15 IU/mL.

### Statistical analysis

The software R3.2.1 was used to generate heat maps and for cluster analysis. The Simpson’s diversity index (D=1−∑(Ni/N)^^2^) was calculated to show the differences in distribution patterns between female and male HCV carriers.

## RESULTS

### Characteristics of HCV genotypes

**Figure 1:**
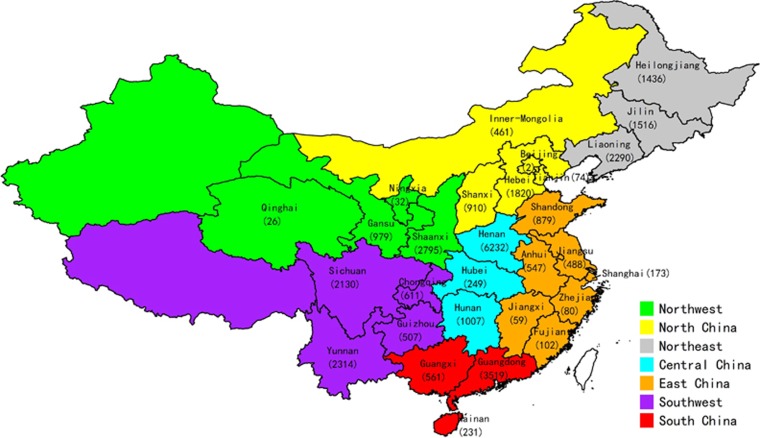
Heatmap of hepatitis C virus (HCV) subtype percentages in 29 provinces. This heatmap was generated using R3.2.1 software. The maximum method was used in clustering analysis.

During the study period, a total of 56 627 serum specimens from 1 900 hospitals located in 29 provinces/municipalities, including Anhui, Beijing, Chongqing, Fujian, Gansu, Guangdong, Guangxi, Guizhou, Hainan, Hebei, Heilongjiang, Henan, Hubei, Hunan, Inner-Mongolia, Jiangsu, Jiangxi, Jilin, Liaoning, Ningxia, Qinghai, Shaanxi, Shandong, Shanghai, Shanxi, Sichuan, Tianjin, Yunnan, and Zhejiang, were obtained for HCV quantification testing by the KingMed Center for Clinical Laboratory. Among the serum specimens, 33 010 met the inclusion criteria and were included in this study, of which 32 030 samples (97.03%) were successfully genotyped/subtyped; however, 980 samples (2.97%) failed the genotyping. The distribution of the samples with positive genotyping results is shown in Figure 1 and is listed in detail in Supplementary Materials S1. Sequencing analysis was done for all rare subtypes. For quality control, sequencing analysis was also applied to confirm the results of the major subtypes, including 394 HCV1b, 35 HCV2a, 6 HCV3a, 53 HCV3b and 120 HCV6a cases. For all samples (major subtypes) that were confirmed by sequencing analysis, the results from the TaqMan analysis and sequencing analysis were consistent.

Ninety-eight of the samples that failed genotyping were also used for HCV quantification with a COBAS AmpliPrep TaqMan kit. Out of these 98 samples, HCV-RNA was not detectable in 91 samples and was detectable at only very low concentrations in 7, whereas the highest copy number obtained was 221 IU/mL. Six hundred and thirty-six samples that were positively genotyped were also positive in the HCV quantitative analysis, and the lowest copy number was 174 IU/mL.

Four genotypes (1, 2, 3 and 6) and 17 subtypes were detected in total. HCV genotype 1 was the most common genotype (*n*=16 799, 52.45%), followed by genotype 2 (*n*=9 193, 28.70%), genotype 3 (*n*=3 736, 11.66%) and genotype 6 (*n*=2 332, 7.28%). Five major subtypes were detected among the 31 659 samples (98.84%): 1b (*n*=16 713, 52.18%), 2a (*n*=9 188, 28.69%), 3b (*n*=2 261, 7.06%), 6a (*n*=2 052, 6.41%) and 3a (*n*=1 479, 4.62%). Mixed infections with the major subtypes were identified in 65 samples (1b–2a, 1b–3b, 1b–6a, 3a–3b, 1b–3a and 2a–6a; *n*=65, 0.20%). Twelve rare subtypes accounting for <1% were identified in this study: 6n (*n*=226, 0.71%), 1a (*n*=86, 0.27%), 6u (*n*=36, 0.11%), 2b (*n*=5, 0.02%), 6g (*n*=4, 0.01%), 6v (*n*=2), 6w (*n*=2), 6b (*n*=1), 6e (*n*=1), 6j (*n*=1), 6q (*n*=1) and 6r (*n*=1). Four subtypes (6b, 6j, 6q and 6r) are reported for the first time in China. Five genotype six strains from Yunnan province could not be subtyped.

**Figure 2:**
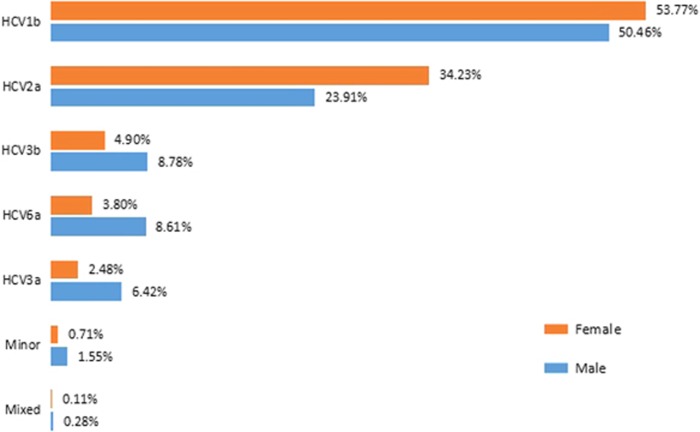
Hepatitis C virus (HCV) subtype distribution of males and females. Minor—minor subtypes; Mixed—mixed infections. The number of male carriers was much greater than females in this study, and male carriers showed a higher level of diversity. The Simpson’s diversity index of male and female carriers was 0.6688 and 0.5892 (*P*=1.23E−234), respectively.

Among the HCV carriers, 17 205 (52.12%) were males, and 14 825 (47.88%) were females. The overall male-to-female ratio was 1.16. Males had a lower percentage of HCV 1b and 2a and a higher percentage of HCV 3b, 6 and 3a and had mixed infections more frequently than females. Male carriers showed higher subtype diversity than females (Figure 2). The median age of male HCV carriers was 48 years, and the median age of female HCV carriers was 52 years.

### HCV genotypes/subtypes and viral load

HCV genotyping/subtyping and HCV viral loading results were obtained for 636 samples. The relationships between viral loads and subtypes were analyzed. The average viral loads among the dominant subtypes were 2.08E+06, 1.88E+06, 3.27E+06, 5.34E+06 and 5.53E+06 for HCV1b, HCV2a, HCV3a, HCV3b and HCV6a, respectively (Table 1). The ranking of average viral load among the subtypes was HCV6a>3b>3a>1b>2a (*P*=7.10E−09). The relevance of viral load within the minor subtypes was not closely examined because of a shortage of cases in this study.

### Geographical distribution of HCV genotypes

**Figure 3:**
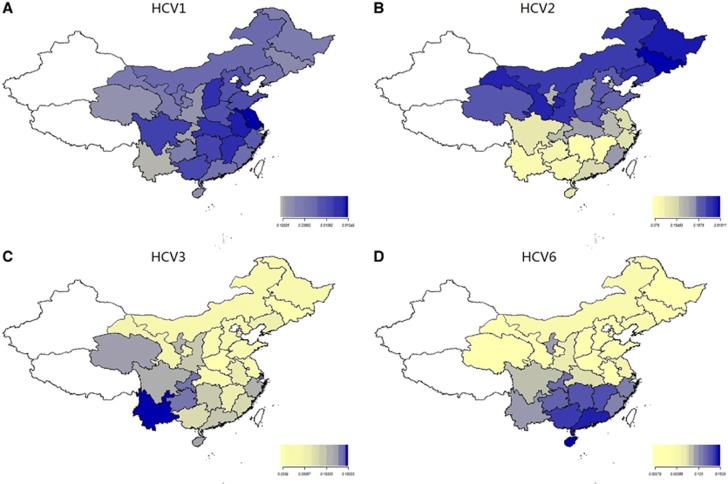
Composition of HCV genotype 1, 2, 3, 6 in Mainland China. (**A**) The distribution of HCV 1 in Mainland China. (**B**) The distribution of HCV 2 in Mainland China. (**C**) The distribution of HCV 3 in Mainland China. (**D**) The distribution of HCV 6 in mainland China. Data for three provinces were not shown because of the lack of positive cases (Xinjiang, Tibet, Beijing).

Four heatmaps for each genotype were generated by using R 3.2.1 (Figures 3A–3D). HCV genotypes 1, 2, 3 and 6 were found in the 28 provinces, and the distribution patterns of each HCV genotype across China differed. HCV genotype 1 was the dominant genotype in most areas of the country, especially in Jiangsu (87.91%), Anhui (77.7%), Shanxi (71.76%), Jiangxi (72.88%), Hubei (71.08%), and Tianjin (70.27%). (Figure 3A and Supplementary Materials S1).

The distribution of genotype 2 gradually decreased from northern to southern China (Figure 3B). HCV2 was the dominant genotype in the northeast (50.93%) but accounted for less than 10% in southwestern (5.23%) and southern China (8.23%). The three provinces in central China showed very different percentages (*P*<0.001) of HCV2 and a decreasing trend from north to south (Henan: 38.88% Hubei: 22.09% Hunan: 0.89%).

The prevalence of HCV genotype 6 gradually increased from northern to southern China (Figure 3D). In northern and northeastern China, HCV6 accounted for less than 1%, whereas in the south, HCV6 constituted 28.0% of all genotypes and was the second most common. The three provinces in central China showed very different percentages (*P*<0.001) of HCV6 and showed an increasing trend from north to south (Henan: 0.21% Hubei: 4.42% Hunan: 21.35%). The seven provinces in eastern China also showed very different percentages of HCV6 and an increasing trend from north to south (Shandong, Anhui and Jiangsu: lower than 2% Shanghai: 8.09% Fujian, Zhejiang and Jiangxi: higher than 16%).

From the southwest to the northeast, the distribution of HCV genotype 3 exhibited a gradually decreasing trend (Figure 3C). The percentage of HCV3 was 38.7% in the southwest, 13.69% in the south, 11.11% in the northwest, and 2.9% in the northeast.

In general, the distributions of HCV genotypes in the southern provinces of China displayed higher diversity than those in the northern provinces. In all of the northern provinces, HCV1b and 2a were the two predominant subtypes and accounted for 86% or more of the total (Supplementary Materials S1 and Figure 2), whereas in the southern provinces, HCV3b and 6a represented the most substantial proportions of the HCVs.

### Distributions of HCV subtypes

The distributions of subtypes of HCV genotype 1, 2, and 3 are shown in Table 2. For HCV genotype 1, HCV1b was the dominant subtype, and HCV1a was found mainly in southern (1.97%), northwestern (0.98%), and southwestern China (0.82%); Hainan (8.70% south China) and Chongqing (4.88% southwest) had the highest prevalence of HCV1a in this study. For HCV genotype 2, HCV2a was the dominant subtype, and HCV2b was found mainly in Shanghai (5.88%) and Fujian (4.17%). For HCV genotype 3, subtype 3b was slightly more prevalent than 3a in the northeast, southwest, east, and north of China, whereas in the northwest, 3a was more prevalent than 3b; in southern and central China, the prevalence of 3a and 3b was highly similar.

HCV genotype 6 strains were detected in 2332 samples (7.28%), with the most prevalent being 6a (2045/2332, 87.69%) followed by 6n (226/2332, 9.69%), 6u (36/2332, 1.54%), 6g (4 cases), 6v (2 cases), 6w (2 cases), 6e (1 case), 6b (1 case), 6j (1 case), 6q (1 case), 6r (1 case), and 5 strains that could not be subtyped (Supplementary Materials S1). In most provinces, 6a was the most common subtype of the HCV6 genotypes, whereas in Yunnan, 6n and 6a represented 63.6% and 23.6%, respectively. In Sichuan and Guizhou, two provinces adjacent to Yunnan, 6n was rare and accounted for only 8.5% and 4.0%, respectively. The proportion of 6n cases in eastern China was 10.7%. (Table 3).

### Mixed infections

Mixed infections with two genotypes or subtypes were identified in 65 samples from 13 provinces (Chongqing, Guangdong, Guangxi, Heilongjiang, Henan, Hunan, Inner Mongolia, Jilin, Liaoning, Shaanxi, Sichuan, Tianjin, Yunnan), accounting for 0.203% of all the HCV-positive samples. The majority of combinations of HCV genotype-subtype in the mixed infections were 1b–2a (31 cases, 47.7%), followed by 1b–3b (20 cases, 32.3%), 1b–6a (6 cases, 9.2%), 3a–3b (4, 6.2%), 1b–3a (3 cases, 4.6%) and 2a–6a (1 case, 1.5%). Of the 65 mixed infections, 48 samples (73.84%) were from males, and the other 17 (26.16%) were from females (Supplementary Materials S1). Mixed infections with the rare subtypes were not observed in our study.

## DISCUSSION

Chronic HCV infection is the leading cause of end-stage liver disease and is associated with a 1–5% annual risk of developing hepatocellular carcinoma (HCC) after developing cirrhosis and with an incidence of approximately 10–20% over 20–30 years of HCV infection.^15^ A previous study has shown that elimination of HCV can be achieved in the coming decades with concentrated strategies to improve diagnosis and treatment;^16^ however, there is not yet a comprehensive understanding of HCV, especially concerning genotypes with potential for use in the development of targeted and efficient strategies for HDV elimination.

This study revealed that five subtypes (1b, 2a, 3b, 6a and 3a) comprise 98.67% of the HCV infections in Mainland China, and the data published by others have also shown similar results.^9, 17^ Regional differences in the distribution of HCV genotypes were also demonstrated. HCV1b and 2a comprise the greatest proportion of HCV subtypes in northern China, whereas the subtype composition showed more diversity in southern China. Subtype 1b is dominant in most areas of China. Subtype 2a is common in northern China and dominant in the northeast provinces (that is, Heilongjiang, Jilin and Liaoning), but is much less common in southern China. Subtype 3b is dominant in Shanghai (34.62%) and Yunnan (44.95%). These results may be explained by the unique locations and diversification of the population. Shanghai is located on the north–south border and is well known as a free-trade zone, which results in a complex transient population. Yunnan borders Vietnam, Laos and Burma, and its ethnic minority groups might lead to remarkable diversity. Southeast Asia is one of the most important drug-producing bases worldwide, and southwestern China is an important transit area for drug smuggling.^18^ Thus, the proportion of drug abusers in the south is much higher than in the north. On the basis of previous studies, intravenous drug abuse associated with genotype 3 has become the major risk factor for HCV infection,^19^ thus potentially explaining why 3b is the predominant subtype in Yunnan. Although HCV genotype 6 has been widely reported in Asia,^20^ the prevalence has not been well studied in China. A previous study has demonstrated that HCV genotype 6 has significant genetic diversity and probably contains at least 22 (6a–6v) subtypes.^21^ Our results indicated that HCV genotype 6 is common in the southern parts of mainland China (Hainan, Guangxi, Guangdong, Guizhou, Chongqing, Hunan, Sichuan and Yunnan) and is relatively rare in north China. The current study demonstrated that HCV6a is the dominant genotype 6 subtype in China; however, in Yunnan province, the prevalence of HCV subtype 6n is higher than 6a, in contrast with the general distribution of this subtype. Dashan Hu *et al*^22^ have reported that subtype 1b is dominant in the Guangdong Province from 2004–2011, followed by HCV2a; our data show that HCV6a has already replaced 2a as the second most common subtype with a rate of 24.16%. That the transmission of HCV type 6 primarily by injectable drug use may explain the HCV6 epidemics that have spread throughout southern China.^23^ In addition, transient populations, new modes of transmission and increasing global travel may change the proportion of HCV genotypes in mainland China.

In general, HCV genotyping relies on the amplification of a specific portion of the HCV genome and is followed by a detection step such as restriction fragment length polymorphism analysis, line probe reverse hybridization, TaqMan PCR or sequence analysis. Core/E1 and NS5B are the two most commonly used target regions. Qingxian Cai *et al* (2012) have compared the suitability of the core region and NS5B sequences as the target regions and have concluded that the core region is more suitable for clinical application and has greater amplification sensitivity.^24^ In addition, HCV genome sequencing improves understanding of resistance-associated variants, including those occurring naturally before treatment, those acquired by transmission at HCV infection, and those emerging after virologic failure.^5^ In our practice, all HCV-positive samples (defined by virus load >1000 IU/mL) of HCV subtypes 1b, 2a, 6a, 3a and 3b were successfully genotyped by TaqMan PCR. Validation was conducted by comparison of the TaqMan PCR results with sequence analysis data, and some of the validation data are currently published.^25^ For the other genotypes (that is, 6n, 1a, 6u, 6g, 6w, 6v, 2b, 6b, 6e, 6j, 6q and 6r), sequence analysis of the core/E1 region was used as the target, and all but three samples (3/243) were successfully subtyped. For the three samples that could not be subtyped, phylogenetic analysis showed that they could be grouped to HCV genotype 6 but differed from all the known HCV 6 subtypes (data not shown). These samples might belong to other rare HCV6 subtypes; therefore, further effort toward identification should be performed.

When chronic hepatitis C is diagnosed and hepatic fibrosis is present, anti-viral therapies should be initiated immediately.^26^ Different treatment should be considered for the patients with different HCV genotypes; thus, the HCV genotype must be determined prior to anti-viral therapy.^27^ On the basis of the pivotal clinical trials for standard treatment, the sustainable virologic response (SVR) rate for genotype 1 is 91–100%,^28, 29^ and the SVR for genotype 4 patients is 100%.^30^ The SVR rate for genotype 2 is 95%^31^ and the SVR for genotype 3 is slightly less than for genotype 2.^32^ The SVR for genotype 6 is 96%.^33^

The genotype distribution was also variable with respect to sex and age. Males and females showed different patterns of subtype composition. The overall number of male HCV carriers was greater than females. We examined whether this finding was applicable to all provinces in our surveillance, and we found that the pattern was consistent in 19 provinces but not for the other 3 (Jilin, Anhui and Jiangsu). Another phenomenon observed in this study was that the pattern of subtype composition in male carriers was more diverse than in females; specifically, more cases of genotypes 1b and 2a were detected from females, whereas 3b, 6 and 3a were more likely among males. The median age of female HCV carriers (50 years) was greater than for males (45 years). The age–sex distribution showed that the average age of female carriers was also older than that of males. Different habits and behaviors between sexes may affect the infection routes and transmission outcomes, thus exacerbating differences in the composition of genotypes in various populations.

Mixed infections represented only a small proportion (0.203%) of the HCV infections, of which HCV1b co-infected with other subtypes was predominant (88.9%), followed by 3a–3b (4 cases, 11.1%) and 2a–6b (1 case, 1.5%). No other co-infection combinations were detected in this study. It is not surprising that HCV1b, the predominant subtype in the general population, was present in most of the co-infections. Although the numbers of infections with 2a, 3a and 6a were relatively high in some provinces, we found that HCV1b was more likely to be present in co-infections with other subtypes, whereas the other mixed infections (2a–3b, 2a–3a, 3b–6a and 3a–6a) were very rare. Thus, it seems that other factors might facilitate subtype 1b becoming a major agent in co-infections, and this possibility should be studied further. Most co-infections were seen in males (80.6%), and this phenomenon might share a similar mechanism with more diverse patterns of subtype distribution also observed in males. With respect to mixed inter-infections or co-infections, the mode of transmission, characteristics of the natural infection course, and effect of anti-viral treatment are important issues that should be elucidated in the future.

This communication describes our effort to provide up-to-date, general information regarding HCV genotype distributions among different populations and geographic areas in Mainland China. We feel that our results contribute to the comprehensive body of data in the fields of HCV infection diagnosis, treatment and prevention as well as epidemiological research.

## Figures and Tables

**Table 1:**
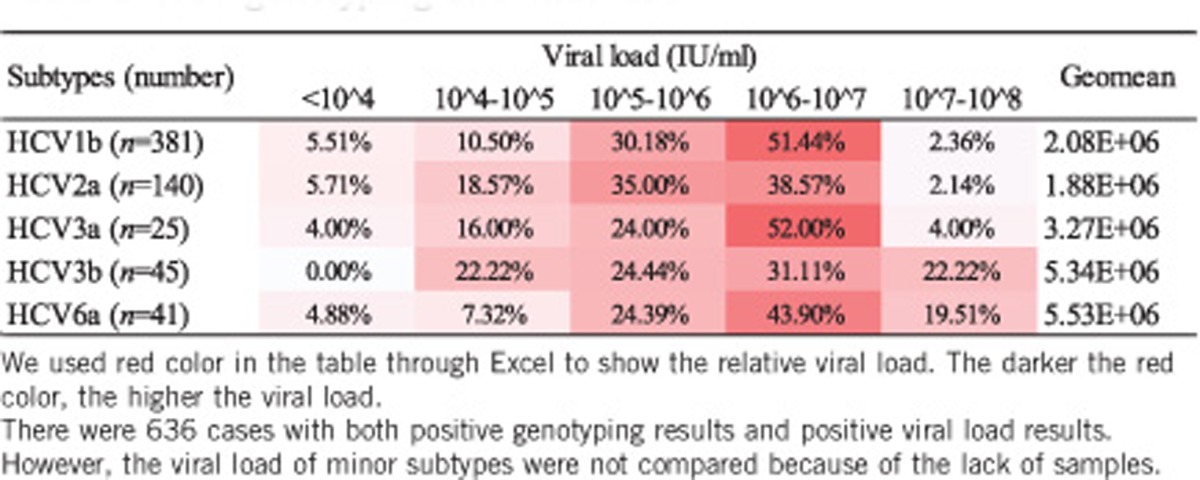
HCV genotyping and viral load

**Table 2:**
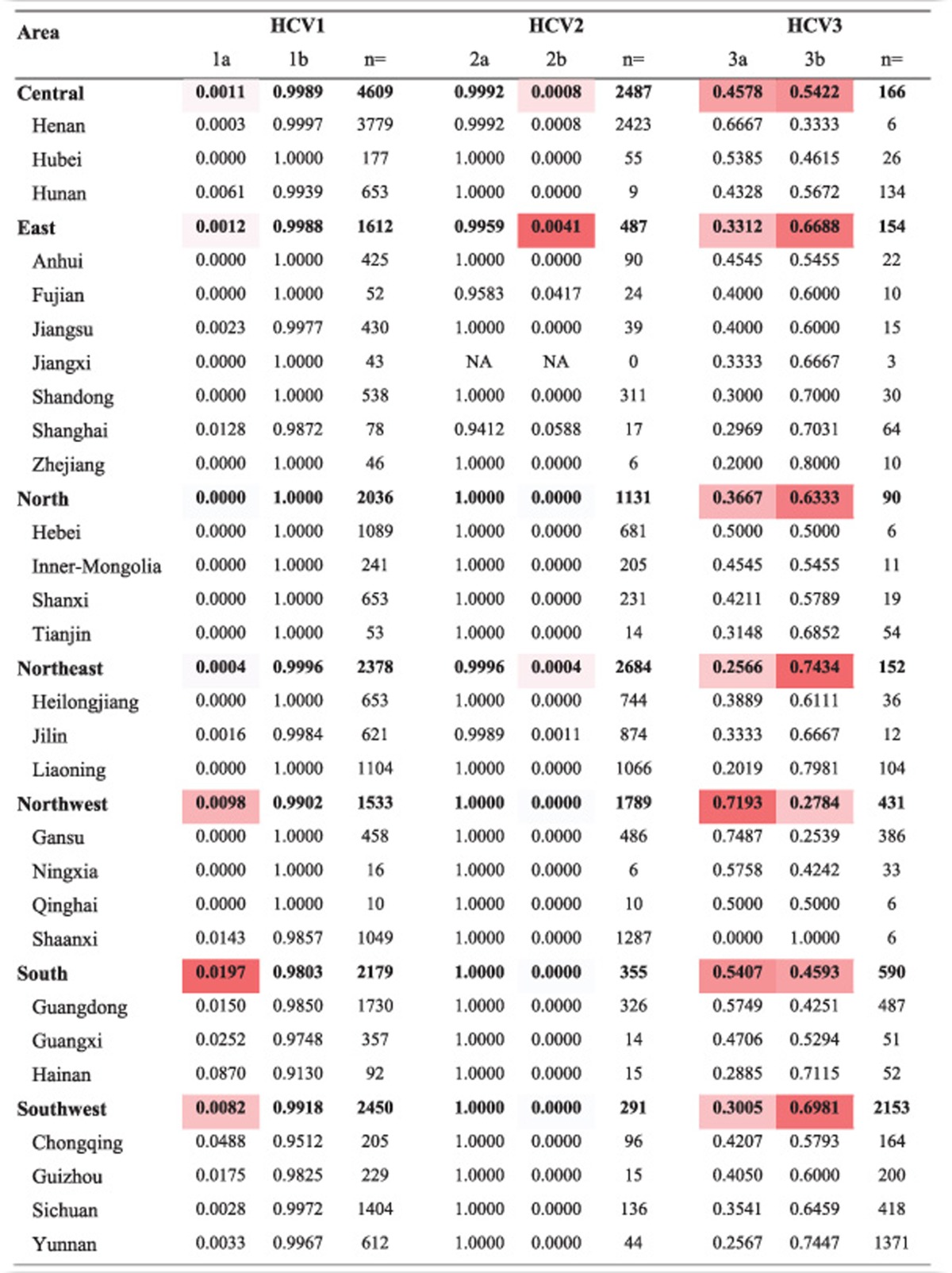
Distribution of subtypes of HCV genotype 1, 2, and 3

**Table 3:** Distribution of HCV 6 subtypes

**Provinces**	**Percentage of the rare subtypes of HCV genotype 6 (%)**													***n*****=**
	**6a**	**6n**	**6u**	**6**	**6g**	**6w**	**6v**	**6xa**	**6b**	**6e**	**6j**	**6q**	**6r**	
**Central**	**100**													**239**
Henan	100													13
Hubei	100													11
Hunan	100													215
**East**	**86.7**	**10.7**				**1.3**		**1.3**						**75**
Anhui	100													2
Fujian	87.5					6.3		6.3						16
Jiangsu	88.9	11.1												9
Jiangxi	100													13
Shandong	87.5	12.5												8
Shanghai	78.6	21.4												14
Zhejiang	76.9	23.1												13
**North**	**94.4**	**5.6**												**18**
Hebei	100													4
Inner-Mongolia	100													5
Shanxi	85.7	14.3												7
Tianjin	100													2
**Northeast**	**92.7**	**7.3**												**41**
Heilongjiang	100													4
Jilin	90.0	10.0												10
Liaoning	92.6	7.4												27
**Northwest**	**92.9**	**3.6**	**1.2**		**1.2**				**1.2**					**84**
Gansu	50.0				50.0									2
Ningxia	100													4
Shaanxi	93.6	3.8	1.3						1.3					78
**South**	**99.1**	**0.3**			**0.2**	**0.2**		**0.1**		**0.1**		**0.1**	**0.1**	**1190**
Guangdong	99.2	0.2				0.2		0.1		0.1		0.1	0.1	979
Guangxi	99.3	0.7												138
Hainan	97.3				2.7									73
**Southwest**	**63.6**	**30.4**	**4.8**	**0.7**			**0.3**				**0.1**			**685**
Chongqing	100													112
Guizhou	96.0	4.0												99
Sichuan	89.8	8.5	1.7											177
Yunnan	23.6	63.6	10.1	1.7			0.7				0.3			297
**Total**	**87.99**	**9.69**	**1.46**	**0.21**	**0.13**	**0.13**	**0.09**	**0.09**	**0.04**	**0.04**	**0.04**	**0.04**	**0.04**	**2332**
