# Supplementary material for: Hepatitis C virus genotypes and subtypes circulating in Mainland China
Source: Emerg Microbes Infect. 2017 Nov 1;6(11):e95–. doi: 10.1038/emi.2017.77 (PMC5717081; doi:10.1038/emi.2017.77)
Supplement: Supplementary Materials S1 [file emi201777x1.pdf]

Supplementary material S1. Number of HCV subtypes identified in 29 provinces

This table shows the number of HCV positive samples (total, female, and male) identified in this study and the percentage of the 5 dominant subtypes in each province.

Number of HCV subtypes identified in 29 provinces

Area	Dominant subtypes					Rare subyptes														Mixed infections						Total
	1b (%)	2a (%)	3b (%)	6a (%)	3a (%)	6n	1a	6u	6	2b	6g	6w	6v	6xa	6b	6e	6j	6q	6r	1b-2a	1b-3b	1b-6a	3a-3b	1b-3a	2a-6a	
**Central**	**4591(61.31)**	**2476(33.07)**	**89(1.19)**	**237(3.17)**	**75(1)**		**5**			**2**										**9**	**1**	**2**		**1**		**7488**
**Henan**	**3769(60.48)**	**2412(38.7)**	**12(0.19)**	**13(0.21)**	**14(0.22)**		**1**			**2**										**9**						**6232**
Female	1853(56.24)	1414(42.91)	3(0.09)	8(0.24)	8(0.24)		1			1										7						3295
Male	1916(65.24)	998(33.98)	9(0.31)	5(0.17)	6(0.2)					1										2						2937
**Hubei**	**177(71.08)**	**55(22.09)**	**2(0.8)**	**11(4.42)**	**4(1.61)**																					**249**
Female	95(76)	25(20)	0	5(4)	0																					125
Male	82(66.13)	30(24.19)	2(1.61)	6(4.84)	4(3.23)																					124
**Hunan**	**645(64.05)**	**9(0.89)**	**75(7.45)**	**213(21.15)**	**57(5.66)**		**4**														**1**	**2**		**1**		**1007**
Female	354(75.32)	2(0.43)	16(3.4)	76(16.17)	20(4.26)		1															1				470
Male	291(54.19)	7(1.3)	59(10.99)	137(25.51)	37(6.89)		3														1	1		1		537
**East**	**1610(69.16)**	**485(20.83)**	**103(4.42)**	**65(2.79)**	**51(2.19)**	**8**	**2**			**2**		**1**														**2328**
**Anhui**	**425(77.7)**	**90(16.45)**	**21(3.84)**	**2(0.37)**	**9(1.65)**																					**547**
Female	240(80.27)	47(15.72)	7(2.34)	2(0.67)	3(1)																					299
Male	185(74.6)	43(17.34)	14(5.65)	0	6(2.42)																					248
**Fujian**	**52(50.98)**	**23(22.55)**	**6(5.88)**	**14(13.73)**	**4(3.92)**					**1**		**1**		**1**												**102**
Female	24(60)	9(22.5)	2(5)	2(5)	2(5)					1																40
Male	28(45.16)	14(22.58)	4(6.45)	12(19.35)	2(3.23)							1		1												62
**Jiangsu**	**429(87.91)**	**39(7.99)**	**8(1.64)**	**8(1.64)**	**2(0.41)**	**1**	**1**																			**488**
Female	228(87.69)	21(8.08)	7(2.69)	1(0.38)	2(0.77)	1																				260
Male	201(88.16)	18(7.89)	1(0.44)	7(3.07)	0		1																			228
**Jiangxi**	**43(72.88)**	**0**	**2(3.39)**	**13(22.03)**	**1(1.69)**																					**59**
Female	22(84.62)	0	1(3.85)	3(11.54)	0																					26
Male	21(63.64)	0	1(3.03)	10(30.3)	1(3.03)																					33
**Shandong**	**538(61.21)**	**311(35.38)**	**12(1.37)**	**7(0.8)**	**10(1.14)**	**1**																				**879**
Female	216(57.6)	151(40.27)	5(1.33)	1(0.27)	1(0.27)	1																				375
Male	322(63.89)	160(31.75)	7(1.39)	6(1.19)	9(1.79)																					504
**Shanghai**	**77(44.51)**	**16(9.25)**	**45(26.01)**	**11(6.36)**	**19(10.98)**	**3**	**1**			**1**																**173**
Female	25(47.17)	10(18.87)	8(15.09)	5(9.43)	4(7.55)	1																				53
Male	52(43.33)	6(5)	37(30.83)	6(5)	15(12.5)	2	1			1																120
**Zhejiang**	**46(57.5)**	**6(7.5)**	**9(11.25)**	**10(12.5)**	**6(7.5)**	**3**																				**80**
Female	19(54.29)	4(11.43)	4(11.43)	6(17.14)	1(2.86)	1																				35
Male	27(60)	2(4.44)	5(11.11)	4(8.89)	5(11.11)	2																				45
**North**	**2029(62.11)**	**1122(34.34)**	**56(1.71)**	**16(0.49)**	**33(1.01)**	**1**														**8**	**1**				**1**	**3267**
**Beijing**	**2(100)**	**0**	**0**	**0**	**0**																					**2**
Male	2(100)	0	0	0	0																					2
**Hebei**	**1081(59.4)**	**673(36.98)**	**37(2.03)**	**4(0.22)**	**17(0.93)**															**8**						**1820**
Female	466(58.84)	311(39.27)	8(1.01)	2(0.25)	5(0.63)																					792
Male	615(59.82)	362(35.21)	29(2.82)	2(0.19)	12(1.17)															8						1028
**Inner-Mongolia**	**241(52.28)**	**204(44.25)**	**6(1.3)**	**4(0.87)**	**5(1.08)**																				**1**	**461**
Female	118(53.39)	98(44.34)	2(0.9)	1(0.45)	1(0.45)																				1	221
Male	123(51.25)	106(44.17)	4(1.67)	3(1.25)	4(1.67)																					240
**Shanxi**	**653(71.76)**	**231(25.38)**	**11(1.21)**	**6(0.66)**	**8(0.88)**	**1**																				**910**
Female	344(70.64)	133(27.31)	5(1.03)	1(0.21)	3(0.62)	1																				487
Male	309(73.05)	98(23.17)	6(1.42)	5(1.18)	5(1.18)																					423
**Tianjin**	**52(70.27)**	**14(18.92)**	**2(2.7)**	**2(2.7)**	**3(4.05)**																**1**					**74**
Female	30(75)	8(20)	1(2.5)	0	0																1					40
Male	22(64.71)	6(17.65)	1(2.94)	2(5.88)	3(8.82)																					34
**Northeast**	**2364(45.1)**	**2670(50.93)**	**113(2.16)**	**38(0.72)**	**39(0.74)**	**3**	**1**			**1**										**13**						**5242**
**Heilongjiang**	**652(45.4)**	**743(51.74)**	**22(1.53)**	**4(0.28)**	**14(0.97)**															**1**						**1436**
Female	334(41.13)	460(56.65)	10(1.23)	2(0.25)	5(0.62)															1						812
Male	318(50.96)	283(45.35)	12(1.92)	2(0.32)	9(1.44)																					624
**Jilin**	**619(40.83)**	**872(57.52)**	**8(0.53)**	**9(0.59)**	**4(0.26)**	**1**	**1**			**1**										**1**						**1516**
Female	327(36.91)	553(62.42)	4(0.45)	1(0.11)	0	1																				886
Male	292(46.35)	319(50.63)	4(0.63)	8(1.27)	4(0.63)		1			1										1						630
**Liaoning**	**1093(47.73)**	**1055(46.07)**	**83(3.62)**	**25(1.09)**	**21(0.92)**	**2**														**11**						**2290**
Female	391(42.41)	490(53.15)	27(2.93)	7(0.76)	4(0.43)	1														2						922
Male	702(51.32)	565(41.3)	56(4.09)	18(1.32)	17(1.24)	1														9						1368
**Northwest**	**1513(39.48)**	**1788(46.66)**	**117(3.05)**	**78(2.04)**	**309(8.06)**	**3**	**15**	**1**			**1**				**1**					**1**	**3**		**1**	**1**		**3832**
**Gansu**	**458(46.78)**	**486(49.64)**	**14(1.43)**	**1(0.1)**	**19(1.94)**						**1**															**979**
Female	236(47.68)	254(51.31)	1(0.2)	0	3(0.61)						1															495
Male	222(45.87)	232(47.93)	13(2.69)	1(0.21)	16(3.31)																					484
**Ningxia**	**16(50)**	**6(18.75)**	**3(9.38)**	**4(12.5)**	**3(9.38)**																					**32**
Female	10(58.82)	3(17.65)	1(5.88)	1(5.88)	2(11.76)																					17
Male	6(40)	3(20)	2(13.33)	3(20)	1(6.67)																					15
**Qinghai**	**10(38.46)**	**10(38.46)**	**6(23.08)**	**0**	**0**																					**26**
Female	6(42.86)	6(42.86)	2(14.29)	0	0																					14
Male	4(33.33)	4(33.33)	4(33.33)	0	0																					12
**Shaanxi**	**1029(36.82)**	**1286(46.01)**	**94(3.36)**	**73(2.61)**	**287(10.27)**	**3**	**15**	**1**							**1**					**1**	**3**		**1**	**1**		**2795**
Female	487(37.18)	743(56.72)	17(1.3)	8(0.61)	55(4.2)																					1310
Male	542(36.5)	543(36.57)	77(5.19)	65(4.38)	232(15.62)	3	15	1							1					1	3		1	1		1485
**South**	**2133(49.48)**	**355(8.23)**	**271(6.29)**	**1176(27.28)**	**319(7.4)**	**3**	**43**				**2**	**2**		**1**		**1**		**1**	**1**			**3**				**4311**
**Guangdong**	**1701(48.34)**	**326(9.26)**	**207(5.88)**	**968(27.51)**	**280(7.96)**	**2**	**26**					**2**		**1**		**1**		**1**	**1**			**3**				**3519**
Female	826(63.01)	181(13.81)	41(3.13)	193(14.72)	55(4.2)		12					1				1			1							1311
Male	875(39.63)	145(6.57)	166(7.52)	775(35.1)	225(10.19)	2	14					1		1				1				3				2208
**Guangxi**	**348(62.03)**	**14(2.5)**	**37(6.6)**	**137(24.42)**	**15(2.67)**	**1**	**9**																			**561**
Female	164(71.3)	5(2.17)	10(4.35)	46(20)	3(1.3)	1	1																			230
Male	184(55.59)	9(2.72)	27(8.16)	91(27.49)	12(3.63)		8																			331
**Hainan**	**84(36.36)**	**15(6.49)**	**27(11.69)**	**71(30.74)**	**24(10.39)**		**8**				**2**															**231**
Female	35(56.45)	8(12.9)	2(3.23)	8(12.9)	6(9.68)		1				2															62
Male	49(28.99)	7(4.14)	25(14.79)	63(37.28)	18(10.65)		7																			169
**Southwest**	**2413(43.38)**	**291(5.23)**	**1488(26.75)**	**435(7.82)**	**646(11.61)**	**208**	**20**	**33**	**5**				**2**				**1**				**15**	**1**	**3**	**1**		**5562**
**Chongqing**	**193(31.59)**	**96(15.71)**	**118(19.31)**	**111(18.17)**	**80(13.09)**		**10**														**1**	**1**	**1**			**611**
Female	85(35.71)	45(18.91)	38(15.97)	43(18.07)	22(9.24)		5																			238
Male	108(28.95)	51(13.67)	80(21.45)	68(18.23)	58(15.55)		5														1	1	1			373
**Guizhou**	**225(44.38)**	**15(2.96)**	**95(18.74)**	**95(18.74)**	**69(13.61)**	**4**	**4**																			**507**
Female	101(44.89)	8(3.56)	44(19.56)	38(16.89)	33(14.67)	1																				225
Male	124(43.97)	7(2.48)	51(18.09)	57(20.21)	36(12.77)	3	4																			282
**Sichuan**	**1395(65.49)**	**136(6.38)**	**266(12.49)**	**159(7.46)**	**147(6.9)**	**15**	**4**	**3**													**4**			**1**		**2130**
Female	709(71.26)	63(6.33)	98(9.85)	79(7.94)	42(4.22)	2	1	1																		995
Male	686(60.44)	73(6.43)	168(14.8)	80(7.05)	105(9.25)	13	3	2													4			1		1135
**Yunnan**	**600(25.93)**	**44(1.9)**	**1009(43.6)**	**70(3.03)**	**350(15.13)**	**189**	**2**	**30**	**5**				**2**				**1**				**10**		**2**			**2314**
Female	227(28.73)	22(2.78)	362(45.82)	24(3.04)	88(11.14)	49	1	11	2												3		1			790
Male	373(24.48)	22(1.44)	647(42.45)	46(3.02)	262(17.19)	140	1	19	3				2				1				7		1			1524
**Total**	**16653(51.99)**	**9187(28.68)**	**2237(6.98)**	**2045(6.38)**	**1472(4.6)**	**226**	**86**	**34**	**5**	**5**	**3**	**3**	**2**	**2**	**1**	**1**	**1**	**1**	**1**	**31**	**20**	**6**	**4**	**3**	**1**	**32030**

*Note: 5 samples collected in Yunnan provice were genotyped as HCV6, and cannot be subtyped by sequence analysis.
